# Fabrication of transparent lead-free KNN glass ceramics by incorporation method

**DOI:** 10.1186/1556-276X-7-136

**Published:** 2012-02-16

**Authors:** Ploypailin Yongsiri, Sukum Eitssayeam, Gobwut Rujijanagul, Somnuk Sirisoonthorn, Tawee Tunkasiri, Kamonpan Pengpat

**Affiliations:** 1 Department of Physics and Materials Science, Faculty of Science, Chiang Mai University, Chiang Mai, 50200, Thailand; 2 Materials Science Research Center, Faculty of Science, Chiang Mai University, Chiang Mai, 50200, Thailand; 3 National Metal and Materials Technology Center, KlongLuang, Pathumthani, 12120, Thailand

**Keywords:** potassium sodium niobate, nanocrystals, ferroelectric, glass ceramics

## Abstract

The incorporation method was employed to produce potassium sodium niobate [KNN] (K_0.5_Na_0.5_NbO_3_) glass ceramics from the KNN-SiO_2 _system. This incorporation method combines a simple mixed-oxide technique for producing KNN powder and a conventional melt-quenching technique to form the resulting glass. KNN was calcined at 800**°**C and subsequently mixed with SiO_2 _in the KNN:SiO_2 _ratio of 75:25 (mol%). The successfully produced optically transparent glass was then subjected to a heat treatment schedule at temperatures ranging from 525**°**C -575**°**C for crystallization. All glass ceramics of more than 40% transmittance crystallized into KNN nanocrystals that were rectangular in shape and dispersed well throughout the glass matrix. The crystal size and crystallinity were found to increase with increasing heat treatment temperature, which in turn plays an important role in controlling the properties of the glass ceramics, including physical, optical, and dielectric properties. The transparency of the glass samples decreased with increasing crystal size. The maximum room temperature dielectric constant (*ε_r_*) was as high as 474 at 10 kHz with an acceptable low loss (tan*δ*) around 0.02 at 10 kHz.

## Introduction

Potassium sodium niobate [KNN] (K_0.5_Na_0.5_NbO_3_) which has a complex perovskite structure was first reported in 1960 by Egerton and Dillon [1]. It also has a high curie temperature of 420**°**C, piezoelectric constant (d_33_) of 80 pC/N, and coupling factor coefficient (k_p_) of 0.35. The crystal structure of KNN is dependent on the temperature [2], where an increase from room temperature to 200**°**C causes an orthorhombic-tetragonal phase transformation, and when the temperature is higher than 420**°**C, the tetragonal phase changes to a cubic phase or becomes paraelectric.

The phase diagram of (1-x) KNbO_3_-xNaNbO_3 _by Jaffe et al. [3] shows that the morphotropic phase boundary of this system occurs at an × approximately 0.5, at which the two orthorhombic phases separate. The as-fired ceramics from this system were found to posses the maximum piezoelectric value even though it is still far from that of the lead-based materials, such as PZT. KNN has recently been subjected to intensive studies as a promising lead-free ferroelectric to replace the toxic PZT.

Ferroelectric glass ceramics were first developed in order to combine the electrical properties of ferroelectric crystals and the transparency of a glass matrix, which makes them suitable for electro-optic applications especially electronic parts, such as electro-optical, high-power lasers, optical integrated circuits, adaptive optics, optical resonator, microwave, and pyroelectric devices [4,5]. KNN ferroelectric glass ceramics have been investigated and attracted much attention since the 1970s [6]. Many research projects have reported that the main problem producing KNN glass ceramics concerns the difficulty in generating the crystallization of the glass ceramics with a pure KNN phase. A secondary phase always occurs in the heat-treated samples.

In this work, the incorporation method was integrated into the glass-ceramic fabrication process. This method modifies the production process by aiming to crystallize only the KNN single phase and reduce the chance of any unwanted second phase which frequently occurred in the conventional method. In this method, starting powders of simple oxides were mixed to form glass batches which could then be subjected to a heat treatment schedule for crystallization, as described in the report of Prapitpongwanich et al. [7]. They also reported that a glass ceramic containing a single LiNbO_3 _phase was achieved in the LiNbO_3_-SiO_2 _system when using the incorporation method. In addition, they were able to make nanocrystals of LiNbO_3 _with improved dielectric property and higher transparency.

In this work, KNN powders were first prepared by calcination, then mixed with SiO_2 _in a Pt crucible, and melted at a suitable temperature. The quench and heat treatment processes were then followed by the crystallization of the KNN crystals, respectively. Here, we report on the physical and electrical properties of the prepared KNN glass ceramics generated using silicate glass modified via the incorporation method. Phase identification, thermal analysis, and microstructures of the prepared glass and glass ceramics were also investigated by X-ray diffraction [XRD], differential thermal analysis [DTA], and scanning electron microscopy [SEM], respectively.

## Materials and methods

**Figure 1:**
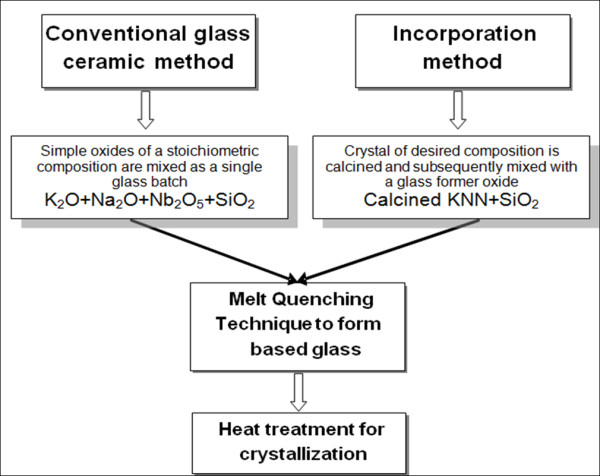
**Comparison between the conventional glass-ceramic method and the incorporation method**.

Figure 1 compared the conventional glass-ceramic method and the incorporation method. In the conventional glass-ceramic method, all simple oxides of a desired composition were melted before being subjected to the heat treatment method for crystallization, while in the incorporation method the calcination or mixed-oxide method was first employed to synthesize the KNN powder before mixing with the glass former oxide which in this case is SiO_2_. To prepare (K_0.5_Na_0.5_NbO_3_) or KNN powder, starting powders (purity > 99%) of K_2_CO_3_, Na_2_CO_3_, and Nb_2_O_5 _were first mixed to form the KNN phase. All compositions were mixed for 24 h, using a wet ball-milling method with alumina balls in a polyethylene bottle. Then, the powders were dried for 24 h and calcined at 800°C in ambient pressure for 2 h. The X-ray diffraction technique was then used to analyze the phase of the calcined powder. Then, the prepared KNN powders were mixed with SiO_2 _in a KNN:SiO_2 _ratio of 75:25 (mol%) using a mortar. Next, the mixture was melted in an electrical furnace at 1,300°C for 1 h. The melt was then quenched between stainless steel plates at room temperature. The prepared glasses were subjected to a heat treatment schedule at the crystallization temperatures ranging from 525°C to 575°C in order to form the glass ceramics with the desired crystal phase. These crystallization temperatures were determined from the DTA trace (Du Pont Instrument, USA) of the as-quenched glass. The density of the glass and glass-ceramic samples were measured employing Archimedes method. The XRD (D500 type, Siemens, UK) and SEM (JSM 6335F type, JEOL, JP) techniques were used to investigate the phase composition and to observe the microstructure of the glass samples, respectively. Two parallel surfaces of the glass ceramics were polished and sputtered with gold as electrodes for the electrical contact. The room temperature dielectric constant (*ε_r_*) and dielectric loss (tan*δ*) of the glass ceramics were measured at various frequencies from 10 kHz to 1 MHz using a precision LCZ meter (E4980A type, Agilent Technologies, Malaysia).

## Results and discussion

**Figure 2:**
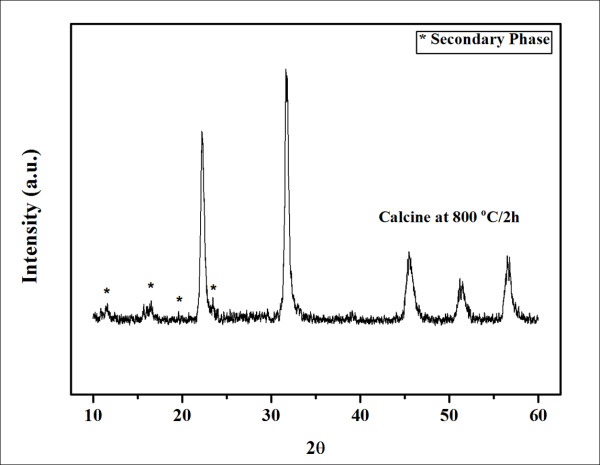
**X-ray diffraction pattern of KNN calcined at 800°C for 2 h**.

The XRD pattern of a calcined KNN sample (Figure 2) displays the diffraction peaks of the KNN perovskite phase, together with an unknown phase. The unknown phase here was K_2_Nb_8_O_21 _(JCPDF 31-1060), which may have occurred from compositional fluctuation during the calcination process. This is in accordance with results reported in the study of Egerton and Dillon and Bomlai et al. [1,8] that the alkaline carbonate precursor was sensitive to moisture, leading to difficulty in obtaining a KNN single phase when using the conventional mixed-oxide technique.

The resulting glass product was light yellow transparent and mechanically robust. To prepare the KNN glass ceramics, many past research projects have used less than 30 mol% of SiO_2 _because it produces a suitable ratio to exhibit transparent regions, therefore, 25 mol% SiO_2 _was chosen to prepare the glass in this work according to Yongsiri et al. [9] However, it has also been reported that the KNN glass with a low content of SiO_2 _possessed low mechanical strength.

**Figure 3:**
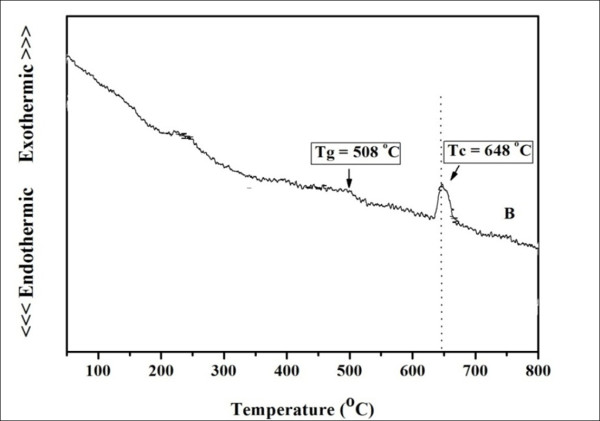
**DTA trace of the as-quenched glass**.

The thermal parameters such as glass transition [T_g_] and crystallization [T_c_] temperatures of the prepared glass were obtained from the DTA trace as shown in Figure 3. The T_g _and T_c _in this system were found to be at about 508°C and 648°C, respectively. The KNN glass was then subjected to a heat treatment schedule at various temperatures from 500°C-648°C to study the crystallization behavior of this glass system. It was found that the glass ceramics subjected to the heat treatment at temperatures higher than 575°C were opaque, while the lower T_c _gave highly transparent glass ceramics. Further investigation has mainly concentrated on those transparent glass-ceramic samples heat treated at lower temperatures.

**Figure 4:**
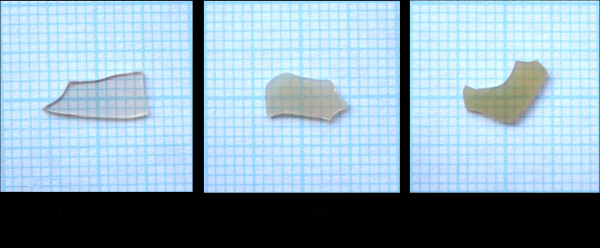
**Appearance of the glass-ceramic samples at various heat treatment [HT] temperatures**.

**Figure 5:**
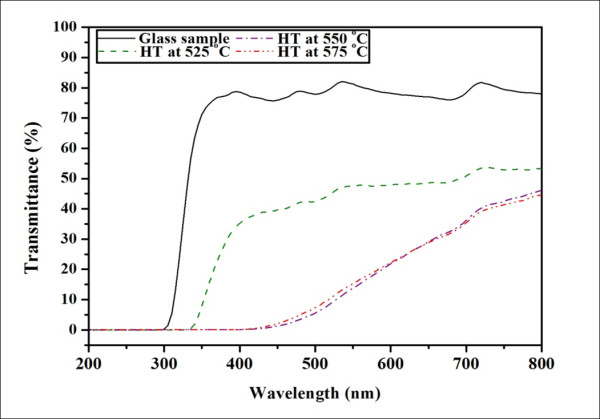
**Percent transmittance of glass-ceramic samples at various HT temperatures**.

The appearances of the prepared glass ceramics are shown in Figure 4. The transparency was found to decrease with increasing temperature of heat treatment. Figure 5 shows the optical transmission spectra recorded at room temperature of the glass and glass ceramics heat treated from 525°C-575°C. The as-quenched glass is optically transparent with nearly 80% transmittance. The transmittance of the heat-treated glass ceramics decreased greatly with increasing heat treatment temperature, while the absorption edges were found to shift toward higher wavelengths. This indicates the change in color from light yellow to brownish yellow of the corresponding glass ceramics. The low transmittance of the glass-ceramic samples may be attributed to the light scattering due to the occurrence of crystals in the sample with sizes larger than 200 nm [10]. Considering the refractive index of the as-quenched glass which is approximately 1.65, and the KNN crystal which is in approximately 2.2, this difference is another factor causing the light scattering at the crystal and glass matrix interface, giving rise to the low transparency.

**Figure 6:**
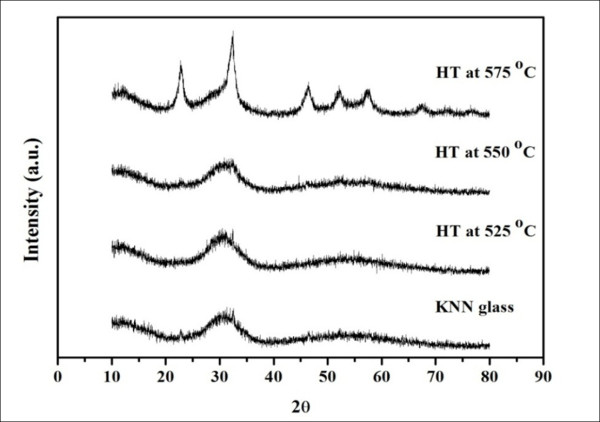
**XRD patterns of glass-ceramic samples at various HT temperatures**.

**Figure 7:**
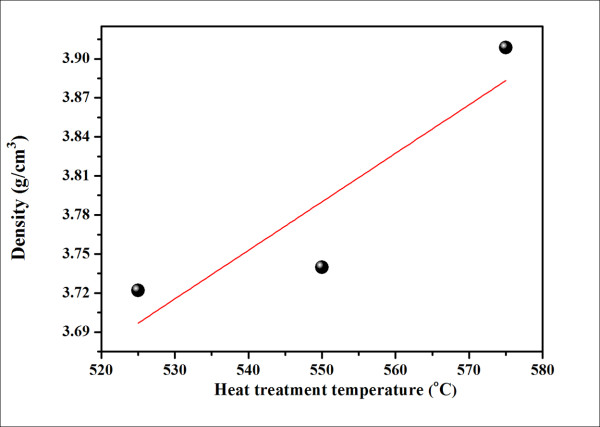
**Density of glass and glass ceramics versus HT temperature**.

Figures 6 and 7 show the XRD patterns and density values of the resulting glass and glass ceramics. As can be seen from the XRD patterns, the samples heat treated from 500°C to 550°C have an amorphous pattern, while that of the 575°C sample contains diffraction peaks of KNN with a highly amorphous phase, giving rise to the lowest transparency of this sample. In Figure 7, the as-quenched glass had the lowest density of about 3.67 g/cm^3^, and the heat treatment caused a general increase in density. It can be assumed that the higher density of these glasses ceramic resulted from the growth of the KNN crystals during the crystallization treatment.

**Figure 8:**
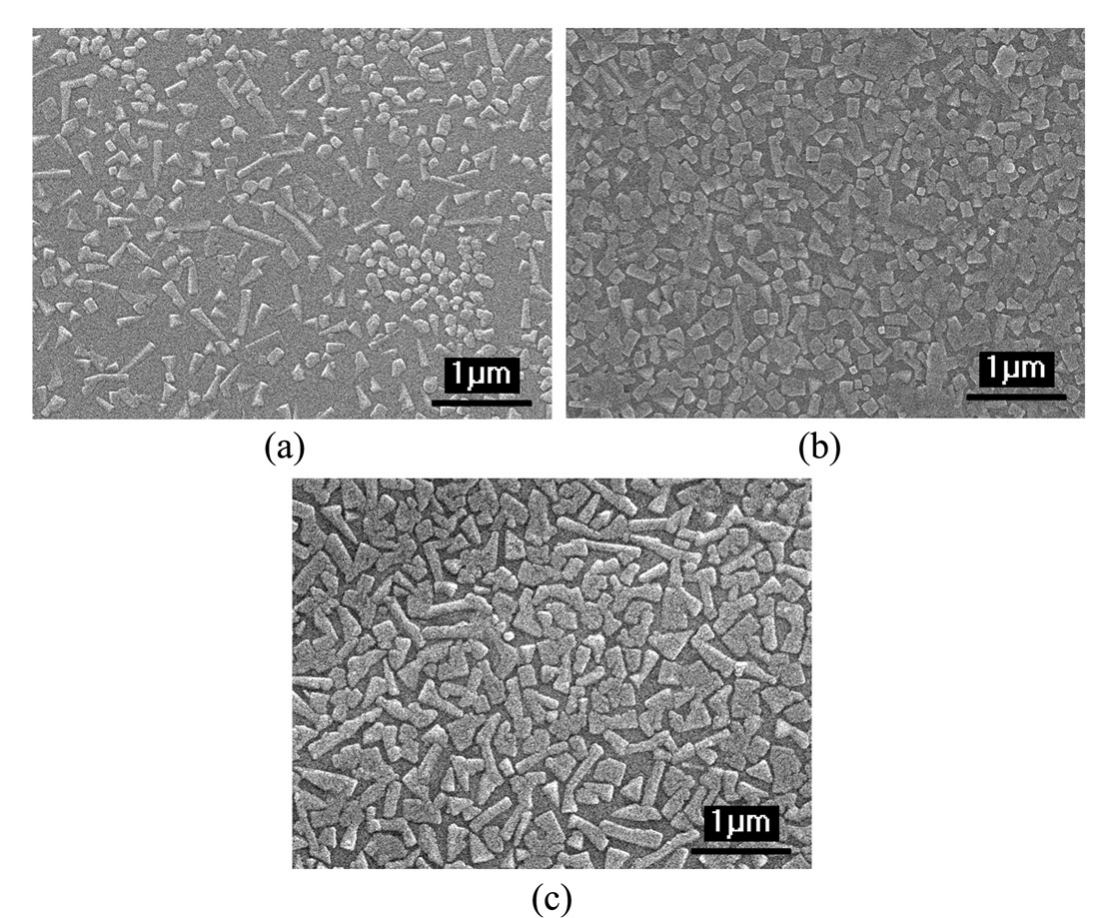
**SEM micrographs of glass-ceramic samples at various HT temperatures**. (a) at 525°C, (b) at 550°C, (c) 575°C (10,000×).

SEM micrographs of the glass ceramics are shown in Figure 8. These micrographs show a bulk crystallization of the KNN phase with a rectangular shape occurred in the glass matrices of all heat-treated samples. Even though, the amorphous XRD patterns were observed in the glass-ceramic samples heat treated at temperatures lower than 550°C, it is clear from the SEM result that crystallization of the KNN phase occurred in all temperatures lower than the observed T_c _of 648°C from the DTA trace. It is likely that the incorporation method using 75 mol% of calcined KNN powder melted together with 25 mol% SiO_2 _of glass former initially stabilized the KNN nuclei, and then, when this glass was subjected to the further heat treatment process, the additional heat was sufficient to generate the crystal growth of the KNN phase.

The average crystal sizes observed from the SEM micrographs are summarized in Table 1. From the micrographs of the samples heat treated at 525°C and 550°C, the KNN crystals were rectangular in shape and were embedded in the glass matrix with random orientations. The diagonal values (*D*) of these crystals are approximately 117 ± 8 nm for 525°C and 145 ± 18 nm for 550°C, which is less than 200 nm, and therefore, affected less light scattering than that of the larger crystals from the glass-ceramic samples heat treated at the higher temperature of 575°C. The cross sections of the KNN crystals in the glass-ceramic samples heated at 575°C have a different morphology than other samples. These crystals lost the rectangular shape and had an average size (*D*) of about 330 nm.

**Table 1:** Summary of crystal morphology and crystallite sizes

Heat treatment temperature: (°C)	Shape		Average crystalline size (nm)
**525**			***L *= 272 ± 224** ***D *= 117 ± 8**
**550**			***L *= 285 ± 234** ***D *= 145 ± 18**
**575**			***L *= 450 ± 61** ***D *= 330 ± 28**

D, diagonal values, L, length.

Considering previous works of Petrovskii et al. and Zhilin et al. [11,12] which studied the phase separation and crystallization in glasses of the Na_2_O-K_2_O-Nb_2_O_5_-SiO_2 _system using the conventional glass-ceramic method, the high temperature of about 700°C was used to precipitate a high-niobate phase which caused small angle light scattering. By using the incorporation method, a lower temperature of 525°C could be used to promote crystallization without any trace of a secondary phase.

**Figure 9:**
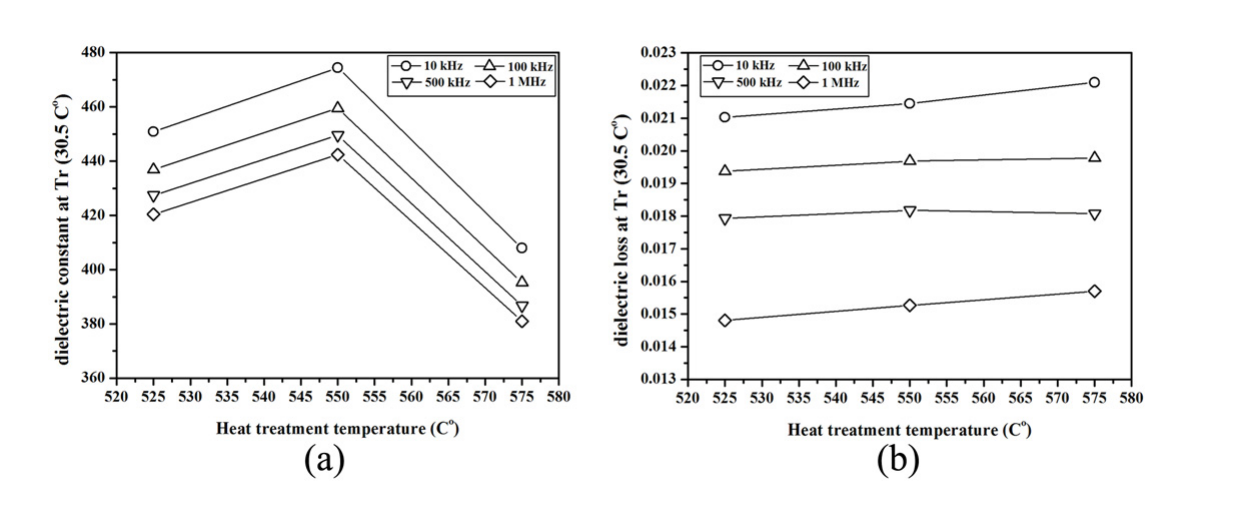
**Dielectric constant (a) and dielectric loss (b) of glass-ceramic samples at various HT temperatures**.

The relationship of the dielectric constant (*ε_r_*) and dielectric loss (tan*δ*) of the glass ceramics with various heat treatment temperatures at 10 kHz to 1 MHz are illustrated in Figure 9ab, respectively. It can be seen that the dielectric constant decreased with increasing heat treatment temperature. The maximum dielectric constant was found in the glass-ceramic sample, heat treated at 550°C, which is as high as 474 at 10 kHz with low value of tan*δ *= 0.02. Considering the SEM micrographs from Figure 8, the sample heat treated at 550°C has higher amount and larger size of KNN crystals than that of the sample heat treated at 525°C, leading to a higher value of dielectric constant of this 550°C heat-treated sample. However, heat treatment at higher temperature such as 575°C caused the reduction in dielectric constant. This may be due to the increase in oxygen vacancies in this sample during heat treatment at the temperature near T_c _of 648°C, at which the high growth rate of KNN crystals occurred. This may attribute to a high atomic migration in the samples heat treated at 575°C, which in turn generates large amount of oxygen vacancies and voids in this glass-ceramic sample.

The overall dielectric loss values of all glass ceramics were low, between 0.014 and 0.023 depending on the frequency. This KNN glass ceramic is a promising lead-free ferroelectric glass ceramic which may be applied to many applications replacing other materials, such as LiNbO_3 _and BaTiO_3 _glass ceramics which have lower dielectric constants. However, further investigation into such properties as the nonlinear optical effects should also be carried out.

## Conclusion

This research shows that heat treatment temperature plays a significant role in controlling the microstructure, crystallite sizes, and crystal quantity of the glass ceramics. Highly transparent KNN glass ceramics can be obtained using the incorporation method with a low heat treatment temperature of 525°C-550°C. The dielectric properties of these glass ceramics are improved, while the transparency value dropped with an increase in the heat treatment temperature. The maximum dielectric constant obtained for these samples was 474 with a low loss (tan*δ*) of 0.02 from the glass-ceramic sample heat treated at 550°C, however, this sample also had the lowest transparency. The optimum values of the dielectric properties in this work promise a bright future for this KNN glass ceramic in electro-optical applications.

## Competing interests

The authors declare that they have no competing interests.

## Authors' contributions

The work presented here was carried out in collaboration between all authors. PY, SE, GR, SS, TT, and KP conceive the research theme. PY and KP designed methods and experiments, carried out the laboratory experiments, analyzed the data, interpreted the results, and wrote the paper. SE, GE, SS, and TT co-designed experiments, discussed, and analyzed the results and presentation. All authors have contributed to, seen, and approved the manuscript.
